# Personal

**Published:** 1899-04

**Authors:** 


### PERSONAL.

DR. SAMUEL GRISWOLD DORR, of Buffalo, has lately been appointed postmaster, to take office April 1, 1899. He is a native of Dansville, N. Y., where his early life was passed. Afterward he lived at Oil Creek and Waterford, Pa. He came to Buffalo in 1873, and entered the medical department of the university from which he received his doctorate degree in 1875. Since that time he has been engaged in the practice of his profession in this city.

Dr. Dorr has been a successful physician and has a large clientèle that both he and his son, Dr. L. Bradley Dorr, minister unto. His appointment as postmaster is a compliment to the profession and is a well-merited honor.

DR. FRANK J. THORNBURY, of Buffalo, has been appointed an assistant surgeon in the U. S. Marine Hospital service, to date from March 9, 1899, and has been assigned to duty at the New York City station. Dr. Thornbury's skill as a bacteriologist will find a useful field in the service that he has entered. His offices in the Colonial, 401 Delaware ave., are for rent. Applications should be made to R. A. Kellogg, 798 Ellicott square.

---

DR. GEORGE RYERSON FOWLER, of Brooklyn, and Dr. A. Walter Suiter, of Herkimer, have been reappointed state medical examiners to fill their own vacancies that will occur August 1, 1899.

---

DR. GROVER W. WENDE, of Buffalo, sailed for Europe by the North German Lloyd line March 22, 1899, to be absent three months at Prague.

---

DR. BENJAMIN FRANKLIN ROGERS, of Buffalo, has returned from the South and resumed his practice in diseases of the eye, ear, nose and throat.

---

DR. HERBERT U. WILLIAMS, of Buffalo, is in Europe for three months, spending most of the time at Göttingen.

